# Phytochemistry and Pharmacological Studies of* Citrus macroptera*: A Medicinal Plant Review

**DOI:** 10.1155/2017/9789802

**Published:** 2017-06-27

**Authors:** Koly Aktar, Tahira Foyzun

**Affiliations:** Department of Pharmacy, Southeast University, Dhaka, Bangladesh

## Abstract

*Citrus macroptera *(family Rutaceae), commonly known as Sat Kara, is a pharmacologically diverse medicinal plant. Various parts of this plant, specifically fruit, have an immense range of medicinal uses in folk medicine directed for a number of ailments. A plethora of active phytochemical constituents of this plant have been revealed so far, namely, limonene, beta-caryophyllene, beta-pinene, geranial edulinine, ribalinine, isoplatydesmine, and so forth. Several studies demonstrated the exploration of pharmacological potential of various parts such as fruits, leaves, and stems of* C. macroptera* as antioxidant, cytotoxic, antimicrobial, thrombolytic, hypoglycemic, anxiolytic, antidepressant, cardioprotective, and hepatoprotective. Furthermore, inhibition of in vitro *α*-amylase, inhibition of paracetamol induced hepatotoxicity, and potentiation of brain antioxidant enzyme are also ascertained. In present review, comprehensive study focused on knowledge regarding several phytopharmacological activities of* Citrus macroptera* has been described.

## 1. Introduction

Since time immemorial, mankind has searched for medicines to remove pain and cure various diseases. Evidence exists for the use of medicinal plants up to 60,000 years ago but more recently, a 5000-year-old Sumerian clay slab was discovered verifying the utilization of medicinal plants for the preparation of drugs [1]. Plants have different chemical compounds like secondary metabolites with many biochemical and bioactivity properties showing applications in various industries such as pharmaceuticals [2–6]. The interest in using natural sources or green medicine or medicinal plants is increasing worldwide due to their safety, efficacy, cultural acceptability, and lesser side effects as compared to synthetic drugs. At present, more than 80% of the global population depends on traditional plant-based medications for treating various human health problems [7–9]. More than 9000 native plants have been identified and recorded for their curative properties [10]. The genus* Citrus* contains many economically important fruits that are grown worldwide for their high nutritional and medicinal value [11].* Citrus* is in the family Rutaceae, which is one of the largest families in order Sapindales. Flowers and leaves of* Citrus* are usually strong scented, the extracts of which contain many useful flavonoids and other compounds that are effective insecticides, fungicides, and medicinal agents [12–14].* Citrus* genus includes some of the most important cultivated fruit trees worldwide [15].* C. macroptera* is a semiwild species of* Citrus* native in Malesia and Melanesia [16]. The* C. macroptera* plant is grown in the places of most homesteads and hill tracts of the Sylhet division of Bangladesh [17]. The fruit of* C. macroptera* has significant cytotoxic, antimicrobial [18], antihypertensive, antipyretic, and appetite stimulant potentials [19, 20]. Additionally, significant hypoglycemic and neuropharmacological effects were confirmed in a rat model [21, 22]. It is reported that stem bark of* C. macroptera* possesses antioxidant activity [23] and essential oil obtained from the leave possesses antimicrobial activities [24]. Literature review suggested that plant extracts having antioxidant activities have health promoting effects and antiaging effects and are used for various metabolic and chronic diseases like cancer, liver diseases, inflammation, diabetes, arthritis, and stroke [25, 26].

However, no comprehensive review of this plant has been reported which demonstrates the efficacy of this plant in all dimensions. The present review is aimed at providing comprehensive and current information regarding the pharmacological potentials of* C. macroptera*.

### 1.1. Taxonomic Classification and Common Names

  Kingdom: Plantae
  Order: Sapindales
  Family: Rutaceae
  Genus:* Citrus*
  Subgenus:* Papeda*
  Species:* C. macroptera*

Common names include Melanesian papeda, wild orange [27], Cabuyao, and Satkara [28].

### 1.2. Introduction to Plant Profile

**Figure 1:**
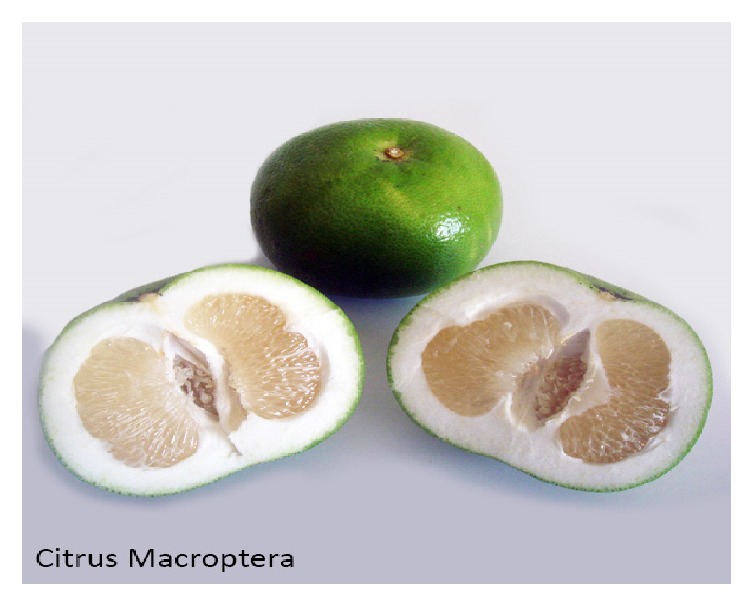
Fruits of* Citrus macroptera*.

The fruit of* C. macroptera *is shown in Figure 1.

## 2. Morphological Studies

*Citrus macroptera* is a semiwild species of* Citrus* genus. It may be mentioned here that the English meaning of Satkara is “wild orange” [29]. Satkara* (C. macroptera)* fruits grow on trees, which are 5 m in height with thorns. The fruit is about 6-7 cm in diameter and the skin is fairly smooth [18]. It is a tree with abundant long spines on the stem, branches, and twigs. The dark green leaves of* C. macroptera* are 2.5–6.8 cm long and 2-3 cm thick. The round or oblong shaped green leaves of this plant are 2.5–3.8 cm in diameter [30]. The shape of fruit is spheroid with concave base and rounded apex and skin color of fruit is yellow with bumpy surface texture. In addition, seeds are of semideltoid shape with wrinkled surface and yellow in color [31].

## 3. Phytochemical Studies

**Figure 2:**
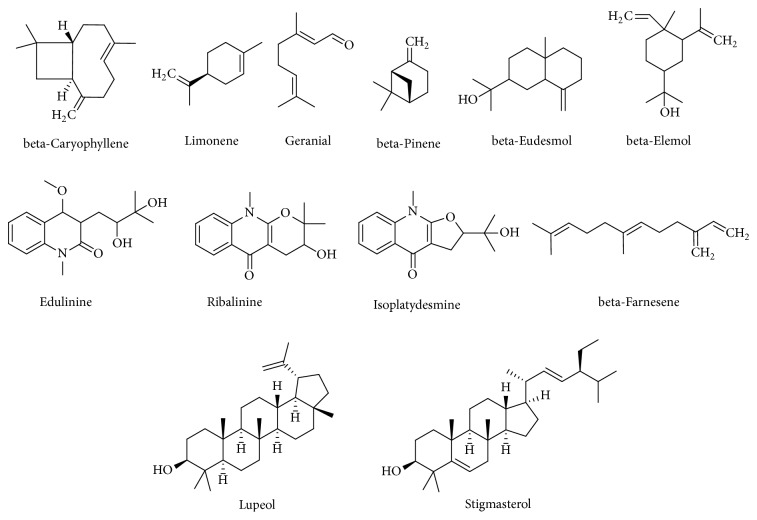
Chemical structures of compounds isolated from* Citrus macroptera.*

Rana and Blazquez (2012) reported that the essential oils obtained by hydrodistillation from the fresh peels of* C. macroptera* contained limonene, beta-caryophyllene, and geranial as main compounds [32]. Jantan et al. (1996) also revealed several phytochemicals from the peels of this plant like monoterpene hydrocarbon, namely, *α*-pinene, *β*-pinene, myrcene, *α*-phellandrene, limonene, and *γ*-terpinene as well as oxygenated monoterpene specially *γ*-elemene, linalool, terpinen-4-ol, *α*-terpineol, terpinolene, and geranyl acetate. Moreover, the peel of this plant has been found to contain some sesquiterpene hydrocarbons, namely, *β*-caryophyllene, (Z)-*β*-farnesene, aromadendrene, and *α*-guaiene, and oxygenated sesquiterpenes including elemol and *β*-eudesmol [33]. Waikedre et al. (2010) showed the beta-pinene as major component of essential oil of* C. macroptera* [24]. Gaillard et al. (1995) isolated edulinine, ribalinine, isoplatydesmine, and five aromatic compounds [34]. Yip and Dallman (1988) and Dallman et al. (1980) also revealed that the phytoconstituents of* C. macroptera*, such as polyphenols, flavonoids, and organosulfur compounds, were found to contribute to its neuropharmacological effects [35, 36]. Chowdhury et al. (2009) isolated two other important chemical compounds, namely, lupeol and stigmasterol [23]. Dreyer and Huey (1973) reported some coumarins like bergamottin, psoralen, marmin, severine, and geiparvarin [37]. However, the chemical structures of several important compounds isolated from different parts of* C. macroptera* are shown in Figure 2.

## 4. Pharmacological Studies

The reported pharmacological activities of various parts of* Citrus macroptera* are detailed below.

### 4.1. Neuropharmacological Activities

The ethanolic extract of* C. macroptera* (EECM) fruit peels was examined for neuropharmacological activities using experimental animal models. Anxiolytic activity was assessed during Elevated Plus Maze (EPM) and Light and Dark Model (LDM) in mice. Acute oral toxicity studies of ethanolic extract of* C. macroptera* (EECM) fruit peels were carried out according to OECD-423 guidelines in mice and it was found to be nontoxic. Ethanolic extract of* Citrus macroptera *(EECM) fruit peels increases number of entries and time spent in light chamber in LDM in mice. Ethanolic extract of* C. macroptera* fruit peels (EECM) was found to possess anxiolytic activities. Moreover, antidepressant activity was assessed using Forced Swim Test (FST) and Tail Suspension Test (TST) in mice. Ethanolic extract of* C. macroptera* fruit peels (EECM) was found to possess antidepressant activities. Furthermore, this was also found to potentiate brain antioxidant enzyme level in experimental animal model [22].

### 4.2. Antioxidant Activities

The methanol and ethyl acetate extract of* C. macroptera* have been extensively studied to evaluate its antioxidant potential by various methods. It has been found that both extracts can scavenge the free radical and stop peroxidation process. The methanolic extract showed more antioxidant potential than ethyl acetate extract in DPPH, NO, and lipid peroxidation assay. However, in cupric acid reducing capacity and lipid peroxidation in human erythrocyte assay the ethyl acetate extract showed more potency than methanol extract [18]. It has also been found that hot methanol extract of stem bark of* Citrus macroptera *showed potential antioxidant activity (IC_50_: 178.96 *μ*g/ml) whereas cold methanol and dichloromethane extracts of the stem bark showed moderate activity with IC_50_ value of 242.78 *μ*g/ml and 255.78 *μ*g/ml, respectively [23].

### 4.3. Paracetamol Induced Hepatorenal Toxicity Inhibition Activity

The ethanolic extract of this plant showed hepato- and nephroprotective activity induced by acetaminophen in rats. The treatment groups of rats were pretreated with extracts for 30 days at 250, 500, and 1000 mg/kg doses after acetaminophen administration. Over the similar period, Silymarin (100 mg/kg) was administered as a standard drug. In contrast to control, lipid peroxidation (TBARS) was increased two times which ultimately marked severe hepatic and renal injuries in association with oxidative stress. Interestingly, histopathological examinations demonstrated that treatment with the plant extract before acetaminophen administration improved all biological parameters studies, that is, transaminase activities, alkaline phosphatase, lactate dehydrogenase, *γ*-glutamyl transferase activities and total bilirubin, total cholesterol, triglyceride and creatinine, urea, uric acid, sodium, potassium and chloride ions, and TBARS levels. This extract showed potential activity at a dose 1000 mg/kg. Thus,* C. macroptera *was suggested to have potential activity probably through lipid peroxidation against acetaminophen-induced hepatonephrotoxicity [38].

### 4.4. Thrombolytic Activity

The methanolic extract of fruits of* C. macroptera *has been reported to possess considerable thrombolytic activity which exerted 48.47% lysis of the blood clot in thrombolytic activity test while 75.18% and 15.82% lysis were obtained in positive control (Streptokinase) and negative control, respectively [39].

### 4.5. In Vitro Cytotoxicity Bioassay

In Brine shrimp lethality bioassay, the methanolic extract of* C. macroptera* fruit showed a LC_50_ value of 30.90 *μ*g/ml compared with the standard vincristine sulphate with LC_50_ value of 10.51 *μ*g/ml [39]. The methanol and ethyl acetate extract of fruit of* C. macroptera* have been considered very toxic where the highest lethality was found for methanol extract which may be due to the presence of steroids, saponins, and terpenoids [18].

### 4.6. Antimicrobial Activities

The antimicrobial activity of methanolic and ethyl acetate extract of* C. macroptera* fruit extract was tested using the disc diffusion technique against six bacterial species where ethyl acetate extract showed broad spectrum antimicrobial activity against two gram positive* Bacillus subtilis* and* Staphylococcus aureus* and one gram negative* Escherichia coli *[18]. The volatile oil extracted from* C. macroptera* fruits was reported to possess reasonable antibacterial activity against four strains including* Bacillus cereus*,* Bacillus subtilis*,* Escherichia coli,* and* Staphylococcus aureus* but not against ampicillin resistant* Escherichia coli* and* Salmonella typhi*. The most potent activity was reported against both* Bacillus* species with a Minimum Inhibitory Concentration of 1.25 mg/ml [40].

### 4.7. Hypoglycemic Activity

The fruit extract of* C. macroptera* revealed moderate *α*-amylase inhibitory activity [IC_50_ value = (3.638 ± 0.190) mg/dl] as compared to acarbose. Moreover, at 500 mg/kg and 1000 mg/kg doses fruit extract significantly (*P* < 0.05 and *P* < 0.01 resp.) reduced fasting blood glucose level in normal rats as compared to glibenclamide (5 mg/kg). In oral glucose tolerance test, 500 mg/kg dose significantly reduced blood glucose level (*P* < 0.05) at 2 h but 1000 mg/kg dose significantly reduced blood glucose level at 2 h and 3 h (*P* < 0.05 and *P* < 0.01 resp.) whereas glibenclamide (5 mg/kg) significantly reduced blood glucose level at every hour after administration. These findings suggest that the plant may be a potential source for the development of new oral hypoglycemic agents [21]. In another study it was revealed that 250 mg/kg dose of methanolic extract of fruit of this plant significantly decreases the level of glycated hemoglobin (*P* < 0.05) leading to the potential hypoglycemic effect of* C. macroptera* [41].

### 4.8. Cardioprotective and Hepatoprotective Activities

An investigation of hematological and biochemical parameters in Sprague-Dawley female rats was performed which evaluated the potent cardioprotective activities of the methanolic extract of* C. macroptera *fruit. It is also claimed that the extract significantly reduced the levels of triglyceride, total cholesterol, low density lipoprotein, and very low density lipoprotein. The extract has also been reported with a significant decrease of alkaline phosphatase and a notable increase of high density lipoprotein cholesterol at a dose of 500 mg/kg and 1000 mg/kg, respectively, which also indicates the cardioprotective and moderate hepatoprotective activities of the fruit extract [41].

## 5. Discussions

The bioactive compounds of plants contain a wide variety of different substances like phytosterols, saponins, phenolic compounds, and terpenoids which have been found to be useful in the prevention and therapy of several diseases, including cancer, and also to have antimicrobial, antifungal, antiparasitic, antiviral antiallergic, antispasmodic, antihyperglycemic, anti-inflammatory, and immunomodulatory properties [42–45]. Encouragingly, current study reviewed several promising pharmacological activities which are intrigued by extensive variety of potential phytoconstituents of* C. macroptera. *

Recently* C. macroptera* was reported to have protective effects against hepatonephrotoxicity [38]. Treatment with paracetamol (APAP) is potentially attributed to hepatic injury and disturbances in the biosynthesis of some enzymes which leak out from the liver cytosol into the blood circulation, thus inducing necrosis and inflammatory responses due to the disruption of hepatocyte membrane permeability by APAP intoxication [46, 47]. This plant was found to maintain membrane integrity and restrict the leakage of hepatic enzymes [38]. Additionally, total bilirubin accumulation is indicative of the binding, conjugation, and excretory capacity of hepatic cells; raised level of it is an important indicator of the severity of necrosis [46, 48]. This plant was reported to restore the above diagnostic marker, thereby displaying its protective role in the liver [38].

Thrombolytic drugs block the pathway of thrombus formation with the help of plasmin that lyses clot by breaking down the fibrinogen and fibrin contained in a clot. Scientists revealed that flavanoids, among the plant metabolites, affect thrombosis and cardiovascular diseases by interfering with platelet activation which is a potential risk factor for cardiovascular disease [49]. In addition, it has been revealed earlier that terpenoids might have significant potentials of demonstrating thrombolytic activity [50, 51]. Admittedly, having abundant flavanoids and terpenoids might lead* C. macroptera* to exhibit thrombolytic activity.

Phytoconstituent showing toxicity is a major concern of scientist and our reviewed plant* C. macroptera* was found to show cytotoxicity which is most often related to chemoprevention. The cytotoxic activity of this plant may be mainly attributed to the compound lupeol which was reported previously as anticancer agent [52].

Some of the phytochemical compounds, for example, glycoside, saponins, tannins, flavanoids, terpenoids, and alkaloids, have been reported to have antimicrobial activity [53]. Several studies revealed that terpenoids are active against bacteria [54–63]. Noteworthy, the reviewed plant contains lupeol which is a pharmacologically active triterpenoid that displayed antimicrobial property earlier [52].* C. macroptera* is profoundly enriched with terpenoids and henceforth may be leading to a remarkable antibacterial activity.

Several investigations revealed hypoglycemic potentials of* C. macroptera.* One study reported that lupeol shows inhibition of activity of *α*-amylase, which hydrolyses complex polysaccharides resulting in increased absorption through small intestine and ultimately enhanced postprandial glucose levels [64, 65]. Furthermore, stem bark of this plant contains two promising phytoconstituents; namely, lupeol and stigmasterol may have hypoglycemic potential. Another study suggested that these two constituents, lupeol and stigmasterol, may act as inhibitor of dipeptidyl peptidase-4 which plays a vital role in glucose metabolism being responsible for the degradation of incretins such as glucagon-like peptide [66, 67]. Moreover, limonene isolated from this plant may be a potential hypoglycemic agent [32].

Earlier, the reviewed plant exhibited potent antioxidant activity. It is well established that oxidative damage to biomolecules (lipids, proteins, and DNA), due to the overproduction of free radical plays an important role in the etiology of numerous diseases such as atherosclerosis, cancer, diabetes, rheumatoid arthritis, post-ischemic perfusion injury, myocardial infarction, cardiovascular diseases, chronic inflammation, stroke and septic shock, aging, and other degenerative diseases in humans [68, 69]. The oxidative stress can be effectively neutralized by enhancing cellular defense in the form of antioxidants which act as a persuasive therapeutic agent against different diseases [70]. Antioxidants exert their effects via several basic mechanisms, which include scavenging the species that initiate peroxidation, quenching singlet oxygen, chelating metals, breaking free radical chain reactions, and reducing the concentration of oxygen [71]. Again recent research suggests that the mechanisms of actions of antioxidants and their role in disease onset or progressions delve deep into cellular signaling processes and control of gene expression [72]. In vivo experiments demonstrated that antioxidants increase both the humoral and the cell-mediated immune response and immune surveillance against tumorigenesis [73–75]. Different studies regarding the potential therapeutic applications of antioxidants in free radical-related diseases led to the hypothesis of their use to slow down or reverse, for example, symptoms associated with neurodegenerative disorders, such as Alzheimer's disease (AD) and Parkinson's disease (PD); such effects could occur through a block of proinflammatory cytokines action and resulting oxidative damage [76–80]. The additional mechanism includes scavenging of a wide range of free radicals including the most active hydroxyl radicals, which initiate lipid peroxidation process [81].

## 6. Conclusion

Presently there is an increasing interest worldwide in herbal medicines accompanied with increased laboratory investigations into the pharmacological properties of the bioactive ingredients and their ability to treat various diseases. Enormous drugs have entered the international market through exploration of ethnopharmacology and traditional medicine. Admittedly,* C. macroptera* can be regarded as a versatile plant having a plethora of medicinal activities. This plant is inimitable source of a wide range of compounds having diverse medicinal properties. The current information regarding this medicinal plant may serve as the baseline data to enforce to do extensive studies for the discovery of new potent compounds and further investigations for their biological activities. Therefore, further research may be carried out on* C. macroptera *to explore their full therapeutic activity.
